# Web-Based Video Education to Improve Uptake of Influenza Vaccination and Other Preventive Health Recommendations in Adults With Inflammatory Bowel Disease: Randomized Controlled Trial of Project PREVENT

**DOI:** 10.2196/42921

**Published:** 2023-08-23

**Authors:** Millie D Long, Welmoed K van Deen, Laura Weisbein, Carine Khalil, Keren L Appel, Xian Zhang, Wenli Chen, Lori Zubrod, Robbie Maris, Afsoon Ghafari, Taylor Dupuy, Christina Y Ha, Brennan M R Spiegel, Christopher V Almario, Gil Y Melmed

**Affiliations:** 1 Division of Gastroenterology and Hepatology University of North Carolina School of Medicine University of Chapel Hill Chapel Hill, NC United States; 2 Erasmus School of Health Policy and Management Health Technology Assessment Section Erasmus University Rotterdam Rotterdam Netherlands; 3 Division of Health Services Research Cedars-Sinai Los Angeles, CA United States; 4 F. Widjaja Foundation Inflammatory Bowel and Immunobiology Research Institute Karsh Division of Digestive and Liver Diseases Cedars-Sinai Medical Center Los Angeles, CA United States; 5 IBD Partners Patient Powered Research Network Washington, DC United States

**Keywords:** preventative, education, inflammatory bowel disease (IBD), education, adults, inflammation, disease, risk, infections, bone, cancer, development, patient, interview, intervention, prevention, vaccination, influenza

## Abstract

**Background:**

Patients with inflammatory bowel disease (IBD) are at increased risk of infections, bone fractures, and skin cancers.

**Objective:**

We developed preventive health videos using a patient-centered approach and tested their impact on preventive health uptake.

**Methods:**

Five animated videos explaining preventive health recommendations in IBD were iteratively developed with patient-centered focus groups and interviews. A randomized controlled trial was then conducted in a web-based IBD cohort to test the impact of video- versus text-based educational interventions. The primary outcome was receipt of the influenza vaccine. Secondary outcomes included intention to receive other preventive health services.

**Results:**

Five animated videos were developed with patient input. A total of 1056 patients with IBD were then randomized to receive the video (n=511) or text-only (n=545) interventions; 55% (281/511) of the video group and 57% (311/545) of the text-only group had received their influenza vaccine in the prior year. Immediately after the intervention, 73% (502/683) of patients reported their intention to receive the vaccine, with no difference by the type of intervention (75%, 231/307, for the video group and 72%, 271/376, for the text-only group). The proportion of patients who actually received the influenza vaccine after the intervention also did not differ by messaging type (*P*=.07). The strongest predictor of both intention to receive and actual receipt of the influenza vaccine was prior influenza vaccination. Older age was also associated with a higher likelihood of the intention to receive (age 36-75 years relative to 18-35 years; *P*=.006) and actual receipt (age >75 years relative to 18-35 years; *P*=.05) of the influenza vaccine.

**Conclusions:**

The proportion of patients receiving the influenza vaccine was high in both groups, but there was no difference in receipt of or in the intention to receive preventive health recommendations by type of messaging. Notably, a portion of patients in both groups had intended to be vaccinated but did not ultimately receive the vaccine. Further evaluation of patient-education strategies is warranted to improve preventive health uptake among patients with IBD.

**Trial Registration:**

ClinicalTrials.gov NCT05997537; https://clinicaltrials.gov/ct2/show/NCT05997537

## Introduction

Inflammatory bowel diseases (IBD), including both Crohn disease (CD) and ulcerative colitis (UC), are chronic, relapsing inflammatory disorders of the gastrointestinal tract associated with substantial morbidity [1,2], missed work and school [3,4], and diminished quality of life [5,6]. IBD management often consists of immunosuppressive medications to treat ongoing inflammation. Patients with IBD have an increased risk of influenza [7], pneumonia [8], herpes zoster [9,10], and other infectious complications [11,12], many of which are associated with significant morbidity and mortality [12]. Patients who received corticosteroids are also at increased risk for bone fractures [13-16]. Some medications used in the treatment of IBD increase the risk of melanoma and nonmelanoma skin cancers [17,18]. Importantly, these complications are potentially preventable—either with vaccination, early detection via screening or surveillance, or sun exposure precautions.

Preventive health guidelines in IBD emphasize the importance of appropriate vaccination, bone health prevention strategies, and cancer screening [19]. However, the knowledge and uptake of preventive health recommendations for patients and providers alike is suboptimal [20-26]. This may be due to the lack of awareness of recommendations, misconception of risks associated with immunizations, or skepticism of vaccine efficacy [20]. These barriers illustrate the need for patient education regarding the importance of preventive care uptake.

To address these barriers, we iteratively developed 5 educational videos (Multimedia Appendices 1-5) with patient input and assessed their effectiveness in increasing influenza vaccine update relative to text-only messaging in a randomized controlled trial.

## Methods

### Patient Focus Groups

**Figure 1:**
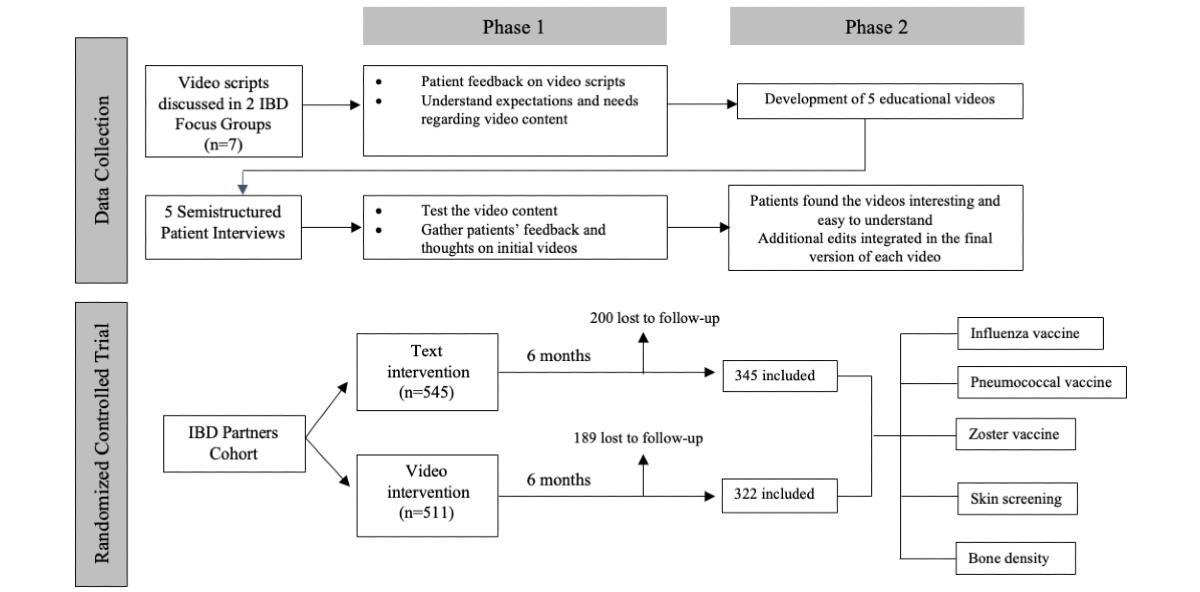
Flow diagram of focus groups and randomized controlled trial. IBD: inflammatory bowel disease.

Scripts were developed for 5 preventive health educational videos (influenza, pneumococcal, and zoster vaccines; bone health; and skin cancer screening) based on best practice guidelines and expert input. To understand patients’ needs and expectations for the videos, we performed two 90-minute focus groups with patients with IBD in which we reviewed and discussed the scripts. We recruited a purposive sample of patients with IBD at Cedars-Sinai Medical Center for 2 in-person focus groups, with a total of 7 patients who had diverse disease experiences in terms of IBD diagnosis, disease duration, and treatments. An inductive thematic analysis was used to analyze users’ needs, preferences, and expectations for the videos (Figure 1).

### Video Development

Based on the insights from the focus groups, the video scripts were adjusted and 5 video prototypes were developed. In 5 semistructured interviews, patient feedback was sought regarding video duration, information visualization, terminology, clarity and consistency of language, and graphics before finalizing the videos. The final product included 5 animated, evidence-based, patient-friendly educational videos (1-2 minutes in length with sound narration).

### Message Development

Text-only messaging reminders included information on indications for vaccination or health screening, complications of these disorders within patients with IBD, recommendations for vaccination or health preventive activities within the IBD population, and references for further reading from the Centers for Disease Control. Patients from the IBD Partners network provided input on the wording and content of the text-based reminders.

### Ethics Approval

The study was reviewed and approved by the institutional review board of Cedars-Sinai Medical Center and University of North Carolina (IRB 19-1607 and 10-0184). All patients provided their informed consent, indicating their willingness to participate in this study. All data are stored and deidentified. Study participants were not compensated for their efforts.

### Randomized Controlled Trial

We next conducted a randomized controlled trial to compare the animated video content (active intervention) with a text-based preventive health reminder for influenza vaccination (control intervention) within the IBD Partners Patient Powered Research Network (PPRN) [27]. Participants were randomly assigned 1:1 using variable block sizes ranging from 4 to 8 stratified by prior year receipt of the influenza vaccine to ensure approximately equal numbers of individuals with no prior influenza vaccination in each group. The study was open to adult patients (aged 18-80 years) with IBD who were members of IBD Partners. Details about the development of the IBD Partners network have been previously published [27,28]. In brief, it is a longitudinal web-based cohort first established at the University of North Carolina in June 2011 by recruiting through the Crohn and Colitis Foundation email rosters, chapter events, and other promotional activities; IBD Partners collects data directly from patients every 6 months. Patients younger than 18 years (who have different childhood vaccination recommendations) and those older than 80 years (who may be more likely to have medical comorbidities that may obviate the need for preventive health measures) were excluded from the study. Participants reporting a prior allergic reaction to the influenza vaccine or who had medical comorbidities that were a contraindication to vaccination were also excluded.

Patients who agreed to participate were assigned to receive 1 of 2 interventions: animated videos or text-only electronic health recommendations via the IBD Partners survey platform. The content of each intervention was targeted to the individual participant’s medical and demographic profile such that only those who met criteria for a specific preventive health recommendation (based on risk factors [age, gender, or steroid or immunosuppressive medication use] and not previously receiving the recommended care) were offered those recommendations.

The primary outcome was patient-reported receipt of the influenza vaccine after the intervention within the 6-month influenza season during which the study was conducted (September 2019 to March 2020). Secondary outcomes were patient-reported intention to receive preventive services and actual receipt of other preventive health measures. The uptake of preventive services was measured as follows [19]: all participants who had not yet received the recommended preventive care were eligible for influenza vaccine and skin cancer education. Those on immunosuppression (biologic, immunomodulator, or small molecule) or aged ≥65 years without prior vaccination were eligible for the pneumococcal vaccine recommendation. Patients aged 50 years or older without prior vaccination were eligible for the shingles vaccine recommendation. At the time of this study, shingles vaccine was not yet recommended for younger age groups on immunosuppression. Those with prior or current corticosteroid use (male or female) or women ≥65 years of age were eligible for the bone health recommendation if they had not previously had a bone density scan. Covariates collected included age, sex, race, education level, disease factors, medication type, and access to primary care. Disease activity was measured using the patient-reported short CD activity index for patients with CD or simple clinical colitis activity index for patients with UC. Patients were invited to participate between September 1, 2019, and March 1, 2020, corresponding with the 2019-2020 flu season. Participants were queried 6 months after enrollment about actual uptake of the influenza vaccine and other preventive health services.

### Sample Size Calculation

Given the baseline rate of influenza vaccination of 65% in the IBD Partners cohort [29], we estimated that the video intervention could result in an improvement of influenza vaccine uptake by 10%. To determine this difference with 90% power and α=.05, we required 460 participants per group for a total n=920.

### Data and Statistical Analysis

We used descriptive statistics to define the characteristics of the study cohort. Continuous variables are described as medians and IQRs. Categorical variables are defined as proportions. The 2 study arms were compared using 2-sided tests of statistical significance (*P*<.05) using the *t* test or the Wilcoxon rank sum test for continuous variables and the chi-square or Fisher exact test for categorical variables.

Stratified analyses were used to assess for intervention-effect heterogeneity based on the following variables: prior immunization history and use of immunosuppressive therapy. Age, sex, smoking status, duration of CD or UC, and current use of immunosuppressive therapy were examined individually for potential confounding of the primary outcome (influenza vaccine uptake) using univariable logistic regression analysis. All variables that affected the crude estimate of the relative risk of effectiveness by 10% or greater were included in the final multivariable model. The study was not powered for subgroup analyses or to detect differences among the other preventive health recommendations (pneumococcal, zoster, bone health, and skin screening); these were considered hypothesis-generating analyses.

## Results

A total of 1056 patients with IBD participated, with 511 randomized to the video intervention and 545 assigned to text-only preventive health messaging (Figure 1). Demographic characteristics including age, gender, race, and education level were similar between the 2 groups (Table 1). Both groups had a female predominance, consistent with the IBD Partners cohort [28]. Disease characteristics were also similar between the 2 groups, including disease duration, disease activity, prior surgery, prior hospitalizations, and current medications (anti–tumor necrosis factor, immunomodulators, corticosteroids, 5-aminosalcylate, anti-integrin, anti–interleukin-12/23, and Janus kinase inhibitors).

Overall, most patients were in clinical remission, defined by a short CD activity index of <150 or a simple clinical colitis activity index of ≤2, including 67% of those receiving the video messages and 62% in the text-based group (Table 1). Similar proportions of patients had received the influenza vaccine in the prior year (58% vs 57%) as well as prior receipt of other preventive health measures, including pneumococcal (53% vs 54%) and herpes zoster (20% vs 24%) vaccination, pap smear (75% vs 77%), dermatology screening (61% in both groups), and bone density screening (57% vs 59%; Table 2).

**Figure 2:**
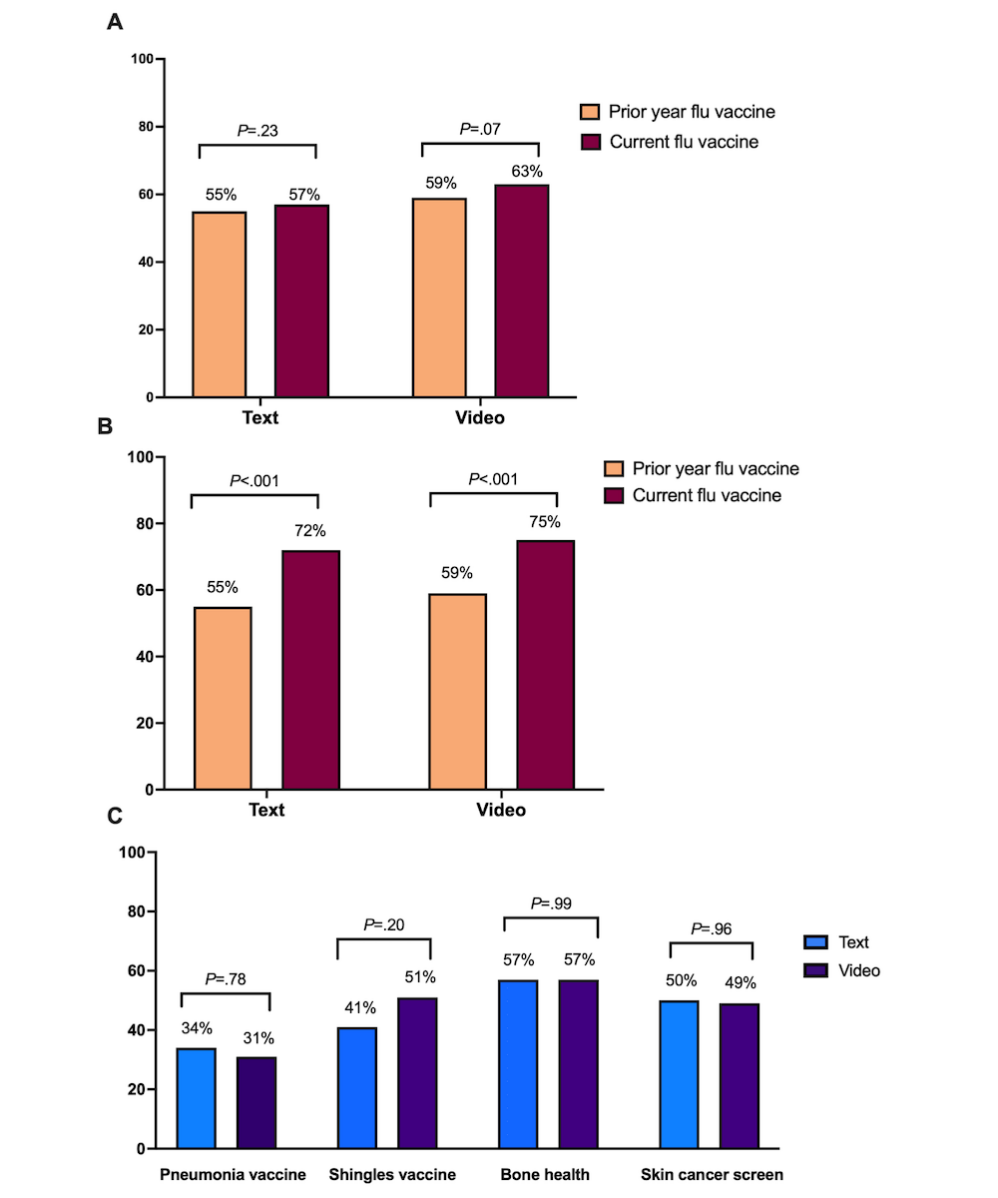
Primary and secondary clinical trial outcomes. (A) No difference in actual influenza vaccine uptake between previous and current years. (B) Increased intent-to-receive flu vaccine after either educational intervention compared with actual receipt of flu vaccine in the prior year. (C) No difference in intent-to-receive other preventive health measures.

With respect to the primary end point, there was no difference in the rate of receipt of the influenza vaccine between the 2 educational interventions in the subsequent flu season. Of the 667 patients who completed the study, 57% of those receiving text education (n=345) and 63% of those receiving video education (n=322) reported receiving the influenza vaccine between September 2019 and March 2020 compared with 55% and 59% in the prior year, respectively (Figure 2A).

A similar proportion of loss of follow-up was noted in both groups for reporting receipt of preventive health measures (Table S1 in Multimedia Appendix 6).

There was no difference in the intention to receive influenza vaccine by receipt of the video versus text intervention immediately after receiving the preventive health recommendation (72% vs 75%, *P*=.62; Figure 2B). There were also no differences between the 2 study arms in intention to receive pneumococcal or shingles vaccines, or bone health or skin cancer screening (Figure 2C; Table S2 in Multimedia Appendix 7).

However, the proportion of patients reporting intention to vaccinate after either intervention (73%) was significantly higher than the proportion of patients who had actually received a vaccine the prior year (57%; *P*<.001). Results were similar when stratified by text (55% prior year actual receipt vs 72% after intention to receive the intervention, *P*<.001) or video intervention (59% prior year actual receipt vs 75% after intention to receive the intervention, *P*<.001; Figure 2B).

We then assessed for the predictors of both intention to receive (Table S3 in Multimedia Appendix 8) and actual receipt (Table 3) of the influenza vaccine using multivariable regression. The strongest predictor of both intention to receive and actual receipt of the influenza vaccine was prior receipt of the influenza vaccine, with those previously receiving the vaccine more likely to currently receive the vaccine. Older age was also associated with a higher likelihood of both intention to receive (age 36-75 years relative to 18-35 years; *P*=.006) and actual receipt (age >75 years relative to age 18-35 years; *P*=.05) of the influenza vaccine. Of the 667 patients included in the final analysis, 63.4% of patients aged 18-35 years (71/112), 58.3% aged 36-75 years (305/523), and 71.9% aged >75 years (23/32) actually received the influenza vaccine after either intervention.

**Table 1:** Patient characteristics at time of enrollment (N=1056).

Characteristic		Text education	Video education	*P* value	
Total number of patients, n		545	511	N/A^a^	
Age (years), mean (SD)		51.2 (14.9)	50.3 (15.6)	.34	
Female gender, n (%)		378 (69)	382 (75)	.05	
**IBD^b^ diagnosis, n (%)**					
	Crohn disease	345 (63)	332 (65)	.57	
	Ulcerative colitis	187 (34)	169 (33)	.67	
	Indeterminate colitis	13 (2)	10 (2)	.63	
High-school education or higher, n (%)		526 (97)	492 (96)	.84	
**Race, n (%)**					.99
	Caucasian	483 (89)	454 (89)		
	African American	8 (1)	7 (1)		
	Other	54 (10)	50 (10)		
Current smoking, n (%)		26 (5)	17 (3)	.24	
BMI, mean (SD)		25.9 (5.8)	25.8 (5.9)	.83	
Disease duration (years), mean (SD)		21.2 (13.5)	20.6 (13.0)	.44	
Ever GI^c^ surgery, n (%)		235 (43)	220 (43)	.98	
Ever GI hospitalization, n (%)		354 (65)	315 (62)	.27	
Number hospitalizations, mean (SD)		3.6 (2.8)	3.4 (2.5)	.48	
**Current medications, n (%)**					
	Immunomodulator^d^	91 (17)	105 (21)	.10	
	Corticosteroids	56 (10)	38 (7)	.11	
	5-ASA^e^	157 (29)	152 (30)	.68	
	Anti-TNF^f^	161 (30)	150 (30)	.99	
	Anti-integrin (vedolizumab)	57 (10)	40 (8)	.16	
	Anti-IL-12/23^g^ (ustekinumab)	42 (8)	37 (7)	.82	
	JAK^h^ inhibitor (tofacitinib)	4 (1)	4 (1)	.92	
Remission (sCDAI^i^<150 or SCCAI^j^≤2), n (%)		297 (62)	296 (67)	.14	
sCDAI, median (IQR)		100 (58-177)	107 (65-170)	.49	
SCCAI, median (IQR)		2 (1-4)	2 (1-3)	.19	

^a^N/A: not applicable.

^b^IBD: inflammatory bowel disease.

^c^GI: gastrointestinal.

^d^Immunomodulator defined as 6-mercaptopurine, azathioprine, or methotrexate.

^e^5-ASA: 5-aminosalicylic acid.

^f^TNF: tumor necrosis factor.

^g^IL: interleukin.

^h^JAK: Janus kinase.

^i^sCDAI: short Crohn disease activity index.

^j^SCCAI: simple clinical colitis activity index.

**Table 2:** Prior vaccination and health maintenance status by intervention at enrollment (N=1056).

Characteristic		Text education	Video education	*P* value	
Total number of patients, n		545	511	N/A^a^	
Flu vaccine in the past year (% yes), n (%)		310 (57)	297 (58)	.68	
**Why not received?, n (%)**					
	Never offered	8 (1)	9 (2)	.70	
	Allergy to eggs or vaccines	10 (2)	9 (2)	.93	
	Did not think I needed it	62 (11)	46 (9)	.20	
	Too expensive	2 (0)	0 (0)	.17	
	Vaccine not available	0 (0)	0 (0)	—^b^	
	Too busy or forgot	25 (5)	21 (4)	.70	
	Concerned about side effects	68 (12)	65 (13)	.91	
	Concerned vaccine would worsen IBD^c^	39 (7)	23 (5)	.07	
	My doctor advised against it	8 (1)	8 (2)	.90	
	Other reason	33 (6)	20 (4)	.11	
	Don’t know	7 (1)	9 (2)	.53	
**Other vaccines (ever) % yes, n (%)**					
	Hepatitis B	285 (52)	266 (52)	.94	
	Hepatitis A	231 (42)	200 (39)	.28	
	Varicella (chicken pox)	166 (31)	158 (31)	.87	
	Pneumococcal (pneumonia)	293 (54)	271 (53)	.81	
	Meningococcal (meningitis)	109 (20)	109 (21)	.59	
	HPV^d^ (cervical cancer and warts)	54 (10)	66 (13)	.12	
	Zoster (shingles)	130 (24)	101 (20)	.11	
	Tetanus	312 (57)	301 (59)	.58	
Had a Pap smear in the last 5 years (women only), n (%)		290 (77)	286 (75)	.55	
**Wear sunscreen**					.43
	Yes, always	65 (12)	64 (13)		
	Yes, most of the time	165 (30)	159 (31)		
	Yes, some of the time	196 (36)	171 (33)		
	No, never	67 (12)	62 (12)		
	Don’t know	0 (0)	3 (1)		
Ever screening skin examination by dermatologist (% yes)		334 (61)	311 (61)	.89	
Ever been told by a doctor that you have osteoporosis or osteopenia (% yes)		200 (37)	188 (37)	.97	
Ever had a bone density study (% yes)		319 (59)	293 (57)	.70	

^a^N/A: not applicable.

^b^—: not available.

^c^IBD: inflammatory bowel disease.

^d^HPV: human papillomavirus.

**Table 3:** Predictive model of factors associated with influenza vaccine receipt (N=667).

Effect	Odds ratio (95% CI)	*P* value
Age (36-75 vs 18-35 years)	0.86 (0.48-1.53)	.61
Age (>75 vs 18-35 years)	3.00 (1.00-8.97)	.049
Immunosuppression (yes vs no)	1.51 (0.97-2.34)	.07
Prior influenza vaccination (yes vs no)	28.49 (18.45-44.00)	<.001
Female (yes vs no)	1.44 (0.89-2.33)	.14
Higher education (yes vs no)	0.78 (0.20-3.03)	.71
Intervention (text vs video)	0.87 (0.57-1.33)	.53

## Discussion

In this study, we developed IBD-focused animated videos for preventive health education with direct patient input and tested their efficacy in a randomized controlled trial using a large web-based cohort. We did not find a difference in the immediate postintervention intention to receive preventive health services between groups of patients receiving text-only versus video interventions, nor did we observe a difference in the actual uptake of these services by messaging type.

Prior studies have assessed the knowledge, attitudes, and beliefs of patients with IBD regarding preventive care using the IBD Partners PPRN [26]. Among individuals who received the influenza vaccine, maintaining health (74%), importance of prevention (66%), and provider recommendation (38%) were the most frequently cited motivations for preventive care uptake [26]. Factors associated with lack of influenza vaccine uptake included lower education level (*P*=.01), younger age (*P*=.02), and no chronic immunosuppression use (*P*<.01) [26]. Additionally, 60% of individuals thought that patients were responsible for keeping track of their vaccines, whereas 45% placed responsibility on their gastroenterologist and 62% on their primary care physician [26]. Among patients who did not receive the influenza vaccine, reasons for nonvaccination included refusal (46%), never offered (12%), allergy (3%), other (26%), and do not know (13%) [26]. The high percentage of patients refusing or never offered vaccination present an opportunity to use patient education to reinforce the importance of preventive care.

The role of text-based electronic reminders emailed to patients was also previously investigated for preventive health recommendations in IBD Partners [29]. In a randomized trial, patients with IBD received either an emailed vaccine educational reminder message or no messaging. A total of 66% of the cohort received an influenza vaccine. Although there was significantly higher overall vaccine receipt among those on immunosuppression, older individuals, and those with a primary care physician (*P*<.001 for each), the vaccination rate among those receiving the email vaccine intervention was no different from that of the placebo arm (66% in both groups, *P*=.94), even when adjusted for those variables.

We were unable to demonstrate an advantage of video-based messaging relative to text-only messages. Short educational videos have previously been associated with higher self-reported IBD patient activation scores and perspective-taking levels (ability to understand thoughts, feelings, and behaviors of patients with IBD) among family and friends [30]. There are also several studies outside of IBD that have shown mixed benefits to video-based patient education [31-35]. Educational videos showed improvement in pneumococcal vaccine uptake in older adults but only when paired with an educational brochure. Other studies showed that educational videos lead to improvements in hemoglobin A_1c_ levels in patients with diabetes mellitus [32], improved satisfaction and comfort in patients undergoing transradial coronary angiography [33], and improved medication compliance in a population with rheumatoid arthritis [34]. However, videos were not successful in improving preoperative anxiety in a pediatric population [35]. Although short videos might change short-term beliefs and satisfaction with care, a more extensive intervention is likely needed to change behavior. This is consistent with our finding that the strongest predictor of future influenza vaccination was prior vaccination, suggesting that patients are set in their beliefs regarding vaccination.

Although it is unlikely that messages conveyed by either text or video will modify knowledge, attitudes, and beliefs regarding preventive health, both text-based and video-based messages may be useful tools to open discussion between physicians and patients. Our video content and text-based messaging are now freely available to providers and patients, including a toolkit for physicians to share with patients and tailored video and text-based messaging to patients based on responses to a short health survey that determines eligibility for specific preventive health services. Notably, a portion of patients in both the video and text-based groups had intended to be vaccinated but did not follow through, suggesting an opportunity to address barriers to vaccination and access to influenza vaccine services.

Although our study findings suggest that video education is not more effective than text-only messages to educate patients with IBD about preventive health, there are potential alternative explanations and limitations to our study. The first consideration is the “ceiling effect,” which occurs when a high proportion of participants already achieve the study objective, so that variance is no longer easily measured above a certain threshold. Several studies in both the adult [20,36,37] and pediatric [38-40] IBD populations have demonstrated lower rates of influenza vaccine uptake than were seen in this study. The uptake of influenza vaccine in the IBD Partners PPRN cohort was already high; therefore, it becomes less likely that any intervention would be significantly better than the standard, text-only intervention, as there is little room for overall measurable improvement in the studied cohort. The lack of racial diversity and overall high level of education among participants may also be a contributing factor as vaccination uptake is generally higher in these populations [41,42].

In summary, we show that vaccine uptake is neither affected by short educational videos nor by short text-based educational interventions in a cohort of highly educated patients with IBD with high baseline vaccination levels. The largest predictor for vaccine uptake is prior receipt of the influenza vaccine, which suggests that future research should specifically focus on patients without prior vaccinations. Further investigation into the optimal means to deliver effective educational messages about preventive health is warranted.

## Appendix

Multimedia Appendix 1 Prevention Bone Health.

Multimedia Appendix 2 Prevention Influenza.

Multimedia Appendix 3 Prevention Pneumonia.

Multimedia Appendix 4 Prevention Shingles.

Multimedia Appendix 5 Prevention Skin Cancer.

Multimedia Appendix 6 Similar loss to follow up between text and video education groups.

Multimedia Appendix 7 Intent to complete prevention interventions immediately after text or video reminders (N=1056).

Multimedia Appendix 8 Predictive model of factors associated with intent to complete influenza vaccine (n=502).

Multimedia Appendix 9 CONSORT-eHEALTH checklist (V 1.6.1).

## Abbreviations

IBD: inflammatory bowel disease
CD: Crohn disease
PPRN: Patient Powered Research Network
UC: ulcerative colitis

## Data Availability

Deidentified data are stored at the University of North Carolina for the IBD Partners cohort.
