# Supplementary material for: Web-Based Video Education to Improve Uptake of Influenza Vaccination and Other Preventive Health Recommendations in Adults With Inflammatory Bowel Disease: Randomized Controlled Trial of Project PREVENT
Source: J Med Internet Res. 2023 Aug 23;25:e42921. doi: 10.2196/42921 (PMC10483303; doi:10.2196/42921)
Supplement: Multimedia Appendix 6 [file jmir_v25i1e42921_app6.docx]

**Table S1.** Similar loss to follow up between text and video education groups

|  | Text Education | Video Education | | | | P value | |  |
| --- | --- | --- | --- | --- | --- | --- | --- | --- |
| Among those receiving flu reminder (all) | 545 | 511 | | | |  | |  |
| Lost follow-up | 200 (37%) | 189 (37%) | | | |  | |  |
| Flu vaccine receipt between Sept 2019 - March 2020 (Yes) | 197 (36%) | 202 (40%) | | | | 0.14 | |  |
| Among those receiving pneumonia vaccine reminder^1^ | 175 | 171 | | | |  | |  |
| Lost follow-up | 63 (36%) | 57 (33%) | | | |  | |  |
| Pneumonia vaccine receipt (Yes) | 12 (11%) | 19 (17%) | | | | 0.19 | |  |
| Among those receiving shingles vaccine reminder^2^ | 195 | 190 | | | |  | |  |
| Lost follow-up | 63 (32%) | 46 (24%) | | | |  | |  |
| Shingles vaccine receipt (Yes) | 13 (10%) | 16 | | | | 0.73 | |  |
| Among those receiving bone health reminder^3^ | 110 | 93 | | | |  | |  |
| Lost follow-up | 38 (35%) | 35 (38%) | | | |  | |  |
| [Bone health screen (Yes)](https://cgibd.med.unc.edu/ccfapartners/researchers/datadic/datadic_queskey.php?queskey=4721) | 4 (6%) | 4 | | | | 0.75 | |  |
| Among those receiving skin cancer screening reminder^4^ | 243 | 240 | | | |  | |  |
| Lost follow-up | 72 (30%) | 84 (35%) | | | |  | |  |
| [Skin cancer screen (Yes)](https://cgibd.med.unc.edu/ccfapartners/researchers/datadic/datadic_queskey.php?queskey=4721) | 12 (7%) | 18 (12%) | | | | 0.16 | |  |
| 1. Patients on immunosuppressive drug or age ≥65 who did not report a prior pneumonia vaccine. |  |  |  | |  | |  | |
| 1. Patients age ≥50 who did not report a prior shingles vaccine. | |  | |  | |  | |  |
| 1. Patients with steroid use and women age ≥65 who did not report a prior bone health screen. | |  | |  | |  | |  |
| 1. Patients who did not report a skin cancer screen within the past year. | |  | |  | |  | |  |
