# Supplementary material for: Web-Based Video Education to Improve Uptake of Influenza Vaccination and Other Preventive Health Recommendations in Adults With Inflammatory Bowel Disease: Randomized Controlled Trial of Project PREVENT
Source: J Med Internet Res. 2023 Aug 23;25:e42921. doi: 10.2196/42921 (PMC10483303; doi:10.2196/42921)
Supplement: Multimedia Appendix 7 [file jmir_v25i1e42921_app7.docx]

**Table S2.** Intent to complete prevention interventions immediately after text or video reminders (N=1056).

| Characteristics | Text education | Video education | |  |  |
| --- | --- | --- | --- | --- | --- |
| Total number of patients | 545 | 511 | |  |  |
| How would you rate the value of the health recommendations presented to you? |  |  | |  |  |
| Total number of patients answered this question | 532 | 450 | |  |  |
| Poor | 14 (3%) | 26 (6%) | |  |  |
| Fair | 36 (7%) | 64 (14%) | |  |  |
| Good | 198 (37%) | 184 (41%) | |  |  |
| Very Good | 176 (33%) | 129 (29%) | |  |  |
| Excellent | 108 (20%) | 47 (10%) | |  |  |
| How likely are you to follow through with any of the health recommendations? |  |  | |  |  |
| Total number of patients answered this question | 530 | 450 | |  |  |
| Not at all | 50 (9%) | 58 (13%) | |  |  |
| Somewhat | 103 (19%) | 95 (21%) | |  |  |
| Likely | 109 (21%) | 99 (22%) | |  |  |
| Very likely | 153 (29%) | 121 (27%) | |  |  |
| Certain | 115 (22%) | 77 (17%) | |  |  |
| Among those receiving flu reminder (all patient) |  |  | |  |  |
| Plan to complete flu vaccine (% yes) |  |  | |  |  |
| Total number of patients answered this question | 376 | 307 | |  |  |
| Yes | 271 (72%) | 231 (75%) | |  |  |
| No | 59 (16%) | 41 (13%) | |  |  |
| Don't know | 46 (12%) | 35 (11%) | |  |  |
| Among those receiving pneumonia vaccine reminder^1^ | 175 | 171 | |  |  |
| Plan to complete pneumonia vaccine (% yes) |  |  | |  |  |
| Total number of patients answered this question | 148 | 124 | |  |  |
| Yes | 50 (34%) | 39 (31%) | |  |  |
| No | 42 (28%) | 40 (32%) | |  |  |
| Don't known | 56 (38%) | 45 (36%) | |  |  |
| Among those receiving shingles vaccine reminder^2^ | 195 | 190 | |  |  |
| Plan to complete shingles vaccine (% yes) |  |  | |  |  |
| Total number of patients answered this question | 172 | 138 | |  |  |
| Yes | 70 (41%) | 70 (51%) | |  |  |
| No | 38 (22%) | 27 (20%) | |  |  |
| Don't know | 64 (37%) | 41 (30%) | |  |  |
| Among those receiving bone health reminder^3^ | 110 | 93 | |  |  |
| Plan to ask doctor about bone health (% yes) |  |  | |  |  |
| Total number of patients answered this question | 97 | 70 | |  |  |
| Yes | 55 (57%) | 40 (57%) | |  |  |
| No | 17 (18%) | 12 (17%) | |  |  |
| Don't know | 25 (26%) | 18 (26%) | |  |  |
| Among those receiving skin cancer screening reminder^4^ | 243 | 240 | |  |  |
| Plan to complete skin cancer screening |  |  | |  |  |
| Total number of patients answered this question | 210 | 188 | |  |  |
| Yes | 105 (50%) | 92 (49%) | |  |  |
| No | 39 (19%) | 37 (20%) | |  |  |
| Don't know | 66 (31%) | 59 (31%) | |  |  |
| 1. Patients on immunosuppressive drug or age ≥65 who did not report a prior pneumonia vaccine. |  |  |  | |  |
| 1. Patients age ≥50 who did not report a prior shingles vaccine. | |  |  | |  |
| 1. Patients with steroid use or women age ≥65 who did not report a prior bone health screen. |  |  |  | |  |
| 1. Patients who did not report a skin cancer screen within the past year. |  |  |  | |  |
