# Supplementary material for: Web-Based Video Education to Improve Uptake of Influenza Vaccination and Other Preventive Health Recommendations in Adults With Inflammatory Bowel Disease: Randomized Controlled Trial of Project PREVENT
Source: J Med Internet Res. 2023 Aug 23;25:e42921. doi: 10.2196/42921 (PMC10483303; doi:10.2196/42921)
Supplement: Multimedia Appendix 8 [file jmir_v25i1e42921_app8.docx]

**Table S3.**Predictive model of factors associated with intent to complete influenza vaccine (n=502).

| **Effect** | **Odds ratio** | **Confidence Interval** | **P value** |
| --- | --- | --- | --- |
| Age (36-75 *vs.* 18-35) | 0.37 | 0.18 – 0.75 | 0.006 |
| Age (>75 *vs.* 18-35) | 0.52 | 0.13 – 2.20 | 0.38 |
| Immunosuppression (Yes*vs*. No) | 1.16 | 0.68 – 1.99 | 0.58 |
| Prior flu shot (Yes *vs.* No) | 37.62 | 16.89 – 83.80 | <0.0001 |
| Female  (Yes *vs.* No) | 0.84 | 0.46 – 1.55 | 0.58 |
| Higher education (Yes *vs.* No) | 0.71 | 0.17 – 3.00 | 0.64 |
| Intervention (Text *vs.* Video) | 0.85 | 0.50 – 1.42 | 0.53 |
